# Relationship between the contact load and time-loss injuries in rugby union

**DOI:** 10.3389/fspor.2024.1395138

**Published:** 2024-09-27

**Authors:** Yusuke Iwasaki, Yuki Someya, Masashi Nagao, Shojiro Nozu, Yuki Shiota, Yuji Takazawa

**Affiliations:** ^1^ Graduate School of Health and Sports Science, Juntendo University, Chiba, Japan; ^2^ Department of Sports Medicine, Juntendo University, Tokyo, Japan; ^3^ Innovative Medical Technology Research & Development Center, Juntendo University, Tokyo, Japan; ^4^ Institute of Health and Sports Science and Medicine, Juntendo University, Chiba, Japan

**Keywords:** contact sports, injury, monitoring, exponentially weighted moving average, acute: chronic workload ratio, GPS

## Abstract

**Objective:**

Quantifying and managing the matches and training loads of players is important for injury prevention. As rugby union is a full-contact sport and frequent contact injuries occur, it might also be important to quantify and manage players’ contact loads. This study aimed to clarify the relationship between contact load and injury incidence in elite rugby union players.

**Methods:**

Forty-eight elite rugby union players (27.0 ± 3.5 years) in Japan were monitored during one season (8 months). The contact load, an index of training load, was evaluated as collision count and collision load measured using a global positioning system device, and then calculated using the acute:chronic workload ratio (ACWR) based on the exponentially weighted moving average (EWMA). The association between the EWMA-ACWR of contact load and injury incidence was analyzed using generalized estimating equations.

**Results:**

Of the 58 injuries during one season, 70.7% were contact injuries. Collision counts and collision load calculated by EWMA-ACWR were associated with the risk of injury (*p* < 0.01 both), with the odds ratios were 4.20 [95% confidence interval (CI): 1.74–10.11] and 4.44 (95% CI: 1.95–10.13), respectively.

**Conclusion:**

Contact load calculated using EWMA-ACWR was associated with injury in elite rugby union players.

## 1 Introduction

Sports injuries are common among athletes. Notably, time-loss injuries prevent players from participating in future training or match play (1), thereby affecting team success in team sports (2, 3). Therefore, strategies to reduce injuries and maximize player availability are crucial. The risk factors for injury incidents are complex and multifactorial and are classified as non-modifiable (e.g., history of previous injury, age, sex, and genetic predisposition) or modifiable (e.g., aerobic fitness, strength, and exposure to loads) (4). Controlling modifiable risk factors is key to preventing injuries in athletes.

A global positioning system (GPS) device was used to measure the overall distance and speed during training sessions or matches (5). Some studies used the acute:chronic workload ratio (ACWR) to quantify and control match and training load in various sports and demonstrated an association between ACWR and injury incidence (6–10). ACWR is the ratio of the acute load to the chronic load, where the short-term load (e.g., the last week) is defined as the acute load and the long-term load (e.g., the previous four weeks) as the chronic load (11). In cricket, Australian football, and rugby league, an ACWR training load of 0.8–1.3 was reported to indicate a low risk of injury, and ACWR > 1.5 was associated with a high risk of injury (11). This theory has been criticized in several papers by Impellizzeri et al., who noted that RA-ACWR is inherently less predictive and introduces statistical artifacts in 2019 and 2021 (12, 13). However, ACWR remains an influential metric in sports science and is used by practice and strength-and-conditioning coaches due to its simplicity and convenience (6). A new method for calculating ACWR, the exponentially weighted moving average (EWMA), which calculates the moving average by assigning a large weight to the most recently undertaken load (14), was recently proposed. Compared with the conventional calculation method of rolling average ACWR (RA-ACWR), EWMA-ACWR showed a greater association with the risk of injuries (15–18).

Rugby union, which is a form of rugby played in teams of fifteen (19), has one of the highest incidences of injury among all professional team sports, with 91 and 2.8 injuries per 1,000 player hours at matches and training, respectively (20). Collisions have been shown to be a significant contributor to the incidence of injuries, with over 60% of all injuries during matches occurring during contact play (20). World Rugby, the international governing body of the rugby union, proposed contact load guidelines in 2021 to manage and limit contact practice time from the perspective of injury prevention (21). Contact load has been evaluated using contact intensity and volume in matches and training (21). Recently, novel GPS devices that enable quantification of the contact intensity and volume have been developed (22) and verified to have a high correlation with video analysis for events identified as collisions (23). As the GPS device can also monitor acute and chronic loads of contact intensity and volume, using EWMA-ACWR in conjunction with novel GPS devices might clarify the risk of injury in rugby union players. Previous studies of rugby union have predominantly focused on non-contact variables such as overall distance measured by GPS (15, 24). However, these studies not sufficiently take into consideration the characteristics of rugby union, which contact plays a critical role, the relationship between the quantification of contact intensity and volume and risk of injury in rugby union remains insufficiently explored. Therefore, this study hypothesized that there is an association between contact load and the risk of injuries in rugby union players, and aimed to investigate the association between the contact load evaluated using GPS devices and time-loss injury in elite male rugby union players by calculating EWMA-ACWR.

## 2 Materials and methods

### 2.1 Study design

This retrospective, observational study used load data evaluated from GPS devices and injury records of 48 elite male rugby union players (mean [standard deviation]; age: 27.5 [3.1] years, height: 180.7 [7.6] cm, body weight: 97.2 [11.8] kg) who belonged to Japan Rugby League One, Japan's professional three-tier rugby union competition (Table 1). All participants were informed of the purpose, methods, procedures, and risks of this study and provided written informed consent. This study was conducted in accordance with the Declaration of Helsinki and was approved by the Ethics Committee for Human Experiments of Juntendo University (No. 2021-115). The observation period covered one season and the pre-season period between August 30, 2021, and May 8, 2022.

**Table 1:** Demographic details of the study participants.

	Total (*n* = 48)	Forwards (*n* = 25)	Backs (*n* = 23)
Age, years	27.5 (3.1)	27.4 (3.3)	27.7 (3.7)
21–25	17 (35.4%)	9 (52.9%)	8 (47.1%)
26–30	20 (41.7%)	11 (55.0%)	9 (45.0%)
31–35	11 (22.9%)	5 (45.5%)	6 (54.5%)
Height, cm	180.7 (7.6)	183.5 (8.1)	177.6 (5.4)
Body weight, kg	97.2 (11.8)	106.0 (7.2)	87.2 (7.1)

Data are expressed as number (%) or mean (standard deviation).

### 2.2 Load data in matches and training

Load data during matches and field-based training sessions were obtained using a GPS device (STATSports Apex, Northern Ireland) (25, 26). This device collected data from a GPS, accelerometer, magnetometer, and gyroscope at frequencies of 10, 952, 10, and 952 Hz, respectively. The GPS device was placed in the small pocket of a specially designed vest and worn on the upper back, that is, over the thoracic spine, between the left and right scapulae. The players wore the same device during the study to eliminate inter-unit variability and errors. Data measured by GPS devices were used to calculate the collision load as contact load, collision count, distance, and high-speed running using STATSports Sonra (STATSports). Collisions were detected by changes in the axis orientation and impact force of >8 g and calculated using a weighted algorithm combining the maximum velocity into the collision, peak impact force, and collision duration (22). Distance and high-speed running data were collected using GPS at a 10 Hz rate; high-speed running was defined as the distance covered at speeds >5.5 m/s (26).

### 2.3 Data processing

ACWR of the collision count, collision load, distance, and high-speed running were calculated for each participant as load indicators in matches and training. The calculation period was 7 days for the acute load and 28 days for the chronic load. In this study, acute and chronic loads were calculated using EWMA. EWMA for any given day was calculated as follows: EWMAtoday=Loadtoday×λa+((1−λa)×EWMAyesterday), where *λ_a_* is a value between 0 and 1, which represents the degree of decay. *λ_a_* was calculated as 2/(*N *+ 1), where *N* is a 7-day (acute) or 28-day (chronic) period. Acute EWMA was then divided by chronic EWMA to provide a single EWMA-ACWR value (14).

### 2.4 Definition of injury

Injury was defined as physical discomfort that occurred during training or a match that prevented full participation in a training session or match and was diagnosed and classified by the team medical staff according to the consensus statement of the International Rugby Board in 2007 (1). Furthermore, the severity (number of days unavailable for training and/or matches), nature of the injury (contact or non-contact), and session in which the injury occurred (training or match) were categorized as previously reported (1).

### 2.5 Statistical analysis

Odds ratios with 95% confidence intervals (CI) were calculated using multiple logistic regression analysis to determine the association between each ACWR and injury occurrence. As this study included repeated ACWR data during the observation period, generalized estimating equations (GEE) were used to model the population-averaged effects of all data. At first, athlete as the subject variable, date of measurement as the within-subject variable, to take into account the correlation between repeated observations of injury incidence within subjects, an autoregressive correlation matrix. The calculate model included injury occurrence (injury/no injury) as the dependent variable, ACWR for each load as the independent variable, position (forward/back), season (pre-season/in-season), and age as confounders. All statistical analyses were performed using SPSS Statistics version 25 (IBM, New York, USA), and statistical significance was set at *P *< 0.05.

After that, the incidence of injury from each ACWR value based on the above results indicated using the following formula (15).

Injury incidence on each ACWR value (per player day) = $\frac{\exp(\mathrm{intercept}+\mathrm{parameter}\;\mathrm{estimate}\times \mathrm{ACWR})}{1+\exp(\mathrm{intercept}+\mathrm{parameter}\;\mathrm{estimate}\times \mathrm{ACWR})}$.

The above calculation was for injuries in forward players.

To calculate the injury incidence in back players, the effect of position was added to the equation as follows: $\frac{\exp((\mathrm{intercept}+\mathrm{parameter}\;\mathrm{estimate}\times \mathrm{ACWR})+\mathrm{position}\;\mathrm{parameter}\;\mathrm{estimate})}{1+\exp((\mathrm{intercept}+\mathrm{parameter}\;\mathrm{estimate}\times \mathrm{ACWR})+\mathrm{position}\;\mathrm{parameter}\;\mathrm{estimate})}$.

## 3 Results

The demographic characteristics of the participants are presented in Table 1. Table 2 shows the total number of injuries, nature of the injury, and session in which the injury occurred. During the cumulative observation period of 9,570 player-days, 58 injuries occurred (5.18 injuries/1,000 player-hours), and the cumulative number of days lost was 1,004 (10.5%). All were categorized as trauma, and 70.7% were contact injuries.

**Table 2:** Total number and nature of injuries according to the session.

	Total	Injury/1,000 player-hours (95% CI)	Contact injury	Non-contact injury
Total number of injuries	58	5.18 (3.9–6.5)	41 (70.7%)	17 (29.3%)
In matches	34	66.7 (43.9–89.4)	29 (85.3%)	5 (14.7%)
In training	24	2.34 (1.42–3.25)	12 (50.0%)	12 (50.0%)

Data are expressed as number (%) or median (95% CI).

**Figure 1:**
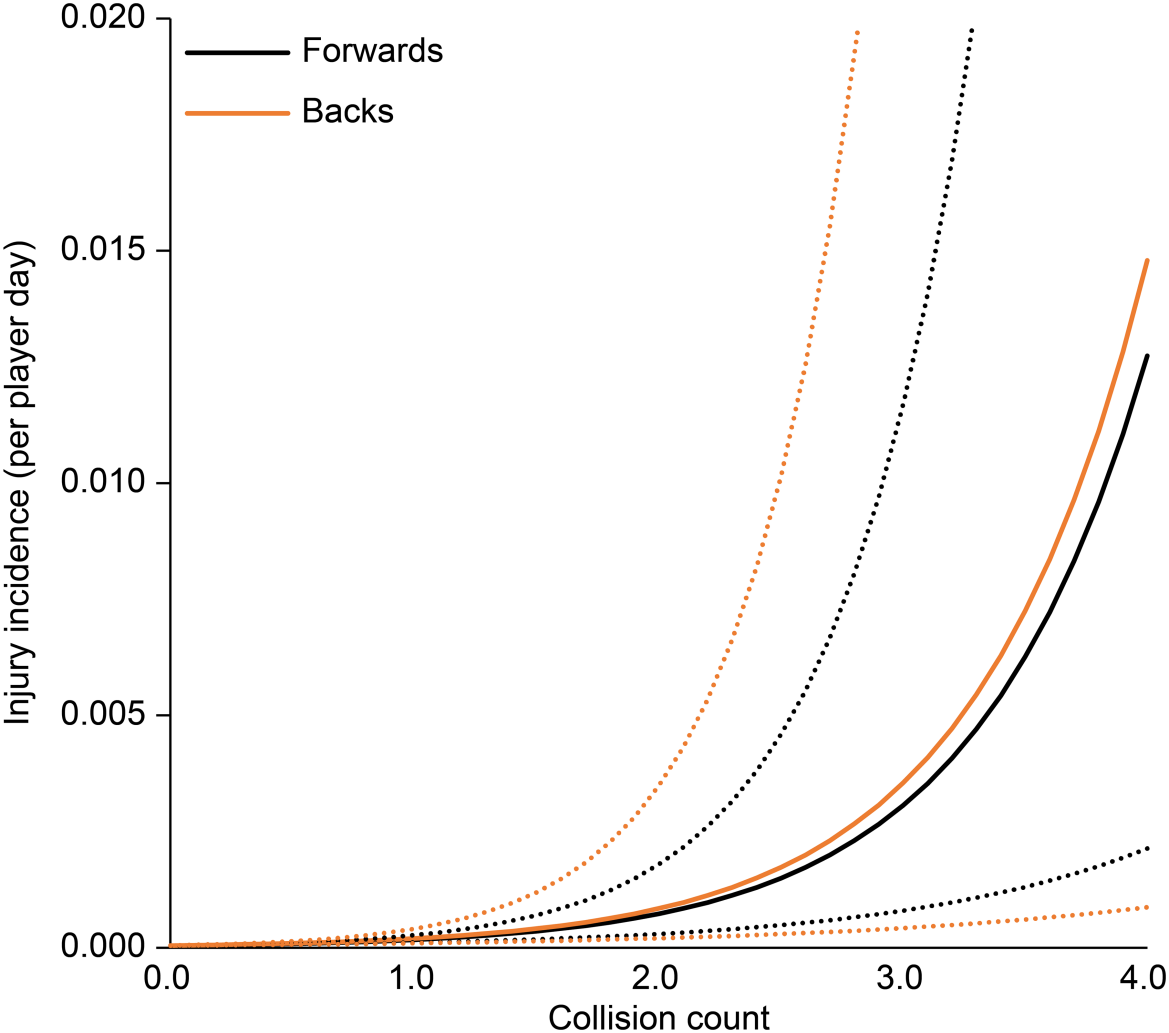
Relationship between collision count (EWMA-ACWR) and injury incidence (per player day). Note: Data are expressed as mean (solid line) and standard error (dotted line). EWMA, exponentially weighted moving average; ACWR, acute:chronic workload ratio.

**Figure 2:**
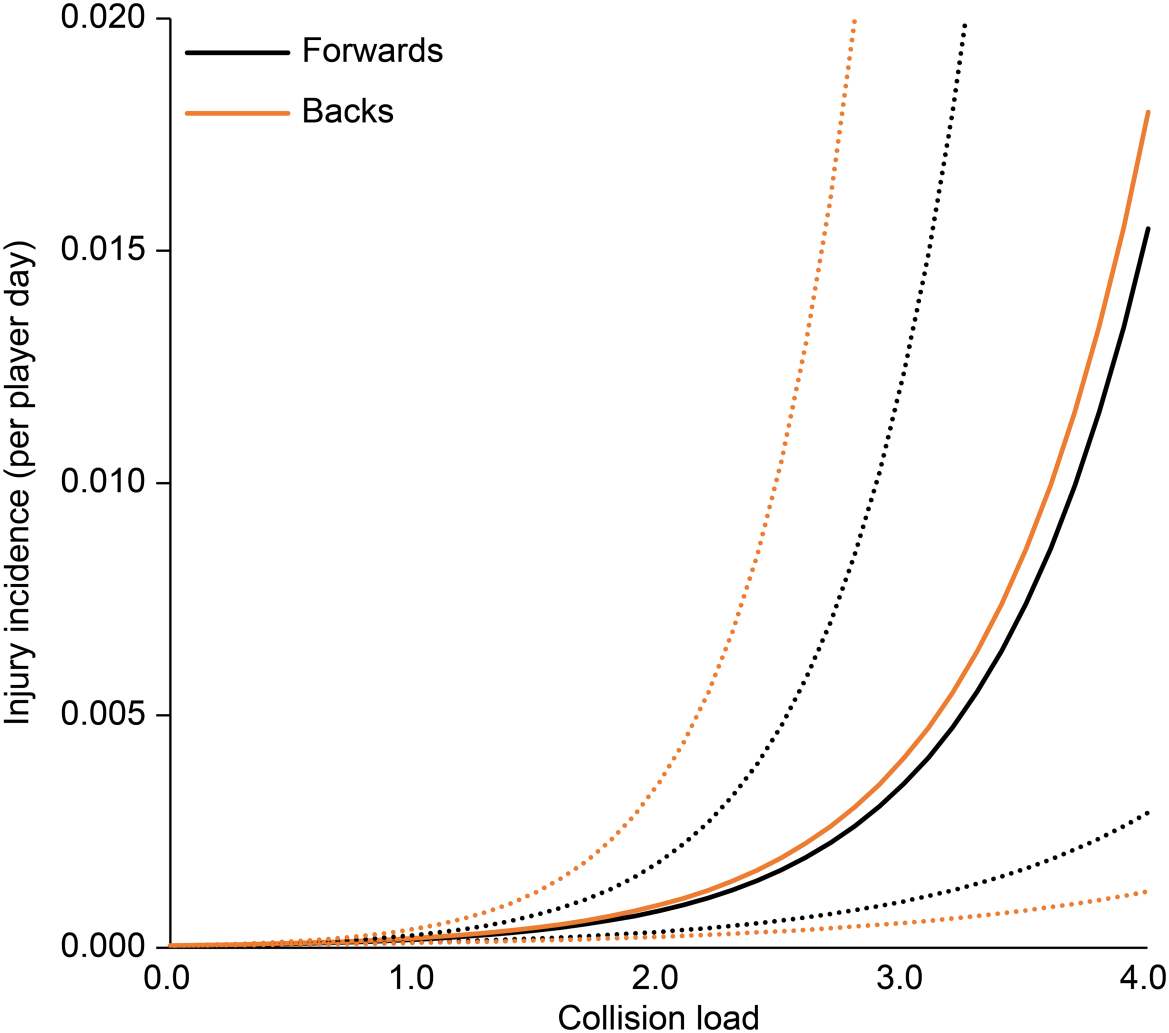
Relationship between collision load (EWMA-ACWR) and injury incidence (per player day). Note: Data are expressed as mean (solid line) and standard error (dotted line). EWMA, exponentially weighted moving average; ACWR, acute:chronic workload ratio.

**Figure 3:**
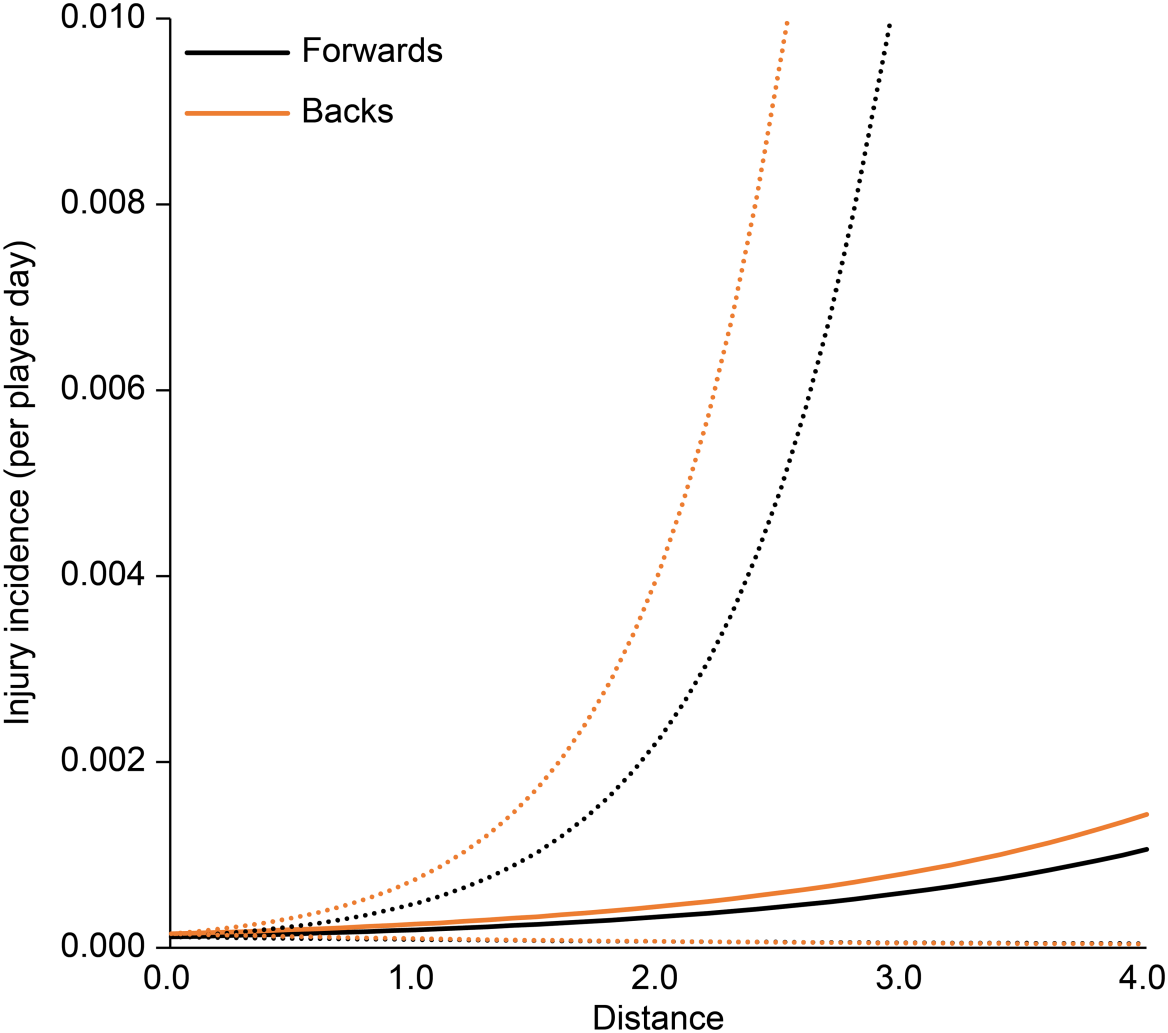
Relationship between distance (EWMA-ACWR) and injury incidence (per player day). Note: Data are expressed as mean (solid line) and standard error (dotted line). EWMA, exponentially weighted moving average; ACWR, acute:chronic workload ratio.

**Figure 4:**
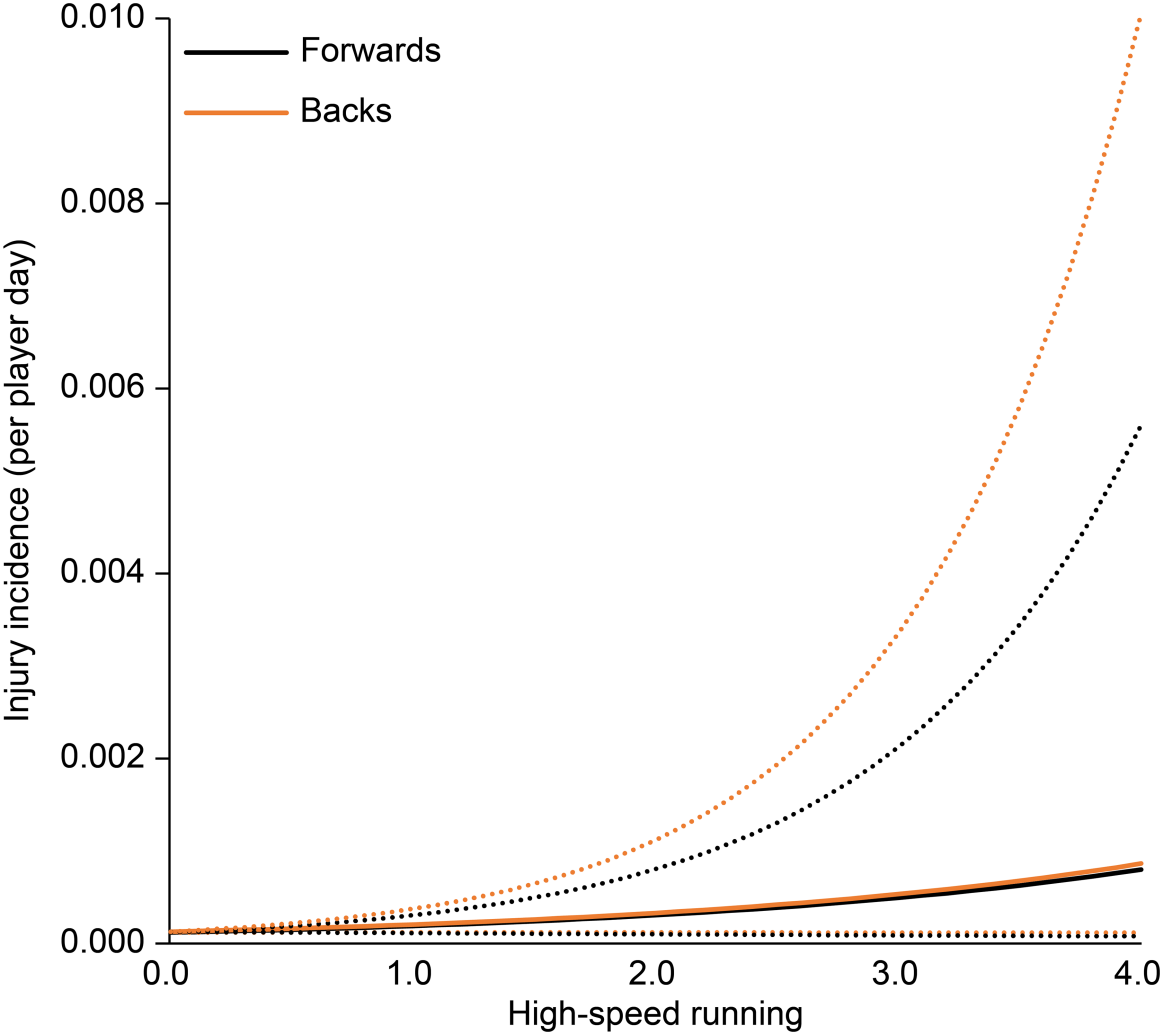
Relationship between high-speed running (EWMA-ACWR) and injury incidence (per player day). Note: Data are expressed as mean (solid line) and standard error (dotted line). EWMA, exponentially weighted moving average; ACWR, acute:chronic workload ratio.

The association between each EWMA-ACWR and the risk of injury is shown in Table 3. In all regression analysis models, age, position, and season were not associated with the risk of injury. The collision count and collision load calculated by EWMA-ACWR were associated with the risk of injury (*p* < 0.01 for both; Figures 1, 2); the odds ratios were 4.20 (95% CI: 1.74–10.11) and 4.44 (95% CI: 1.95–10.13), respectively, for 1 EWMA-ACWR increase in contact load. Distance and high-speed running evaluated by EWMA-ACWR were not associated with the risk of injury (distance: *p* = 0.54; Figure 3; high-speed running: *p* = 0.32; Figure 4).

**Table 3:** Association between each EWMA-ACWR and the risk of injury.

	Intercept	ACWR for each load				Position			Season		
		Parameterestimate	Std. error	Odds ratio	95% CI	Parameter estimate	Odds ratio	95% CI	Parameter estimate	Odds ratio	95% CI
Collision counts	−10.09	1.43	0.45	4.20	1.74–10.11	0.64	1.89	0.47–7.59	0.32	1.37	0.24–7.93
Collision load	−10.12	1.49	0.42	4.44	1.95–10.13	0.64	1.89	0.50–7.13	0.51	1.67	0.33–8.51
Distance	−9.29	0.61	0.99	1.83	0.26–12.75	1.01	2.74	0.30–25.07	0.99	2.70	0.12–63.17
High-speed running	−9.08	0.49	0.49	1.63	0.62–4.24	0.45	1.56	0.47–5.23	0.15	1.16	0.27–4.94

Position is the odds of injury in backs compared to forwards.

EWMA, exponentially weighted moving average; ACWR, acute:chronic workload ratio; CI, confidence interval.

## 4 Discussion

In the present study, we observed 58 injuries (5.2 injuries/1,000 player-hours), 70.7% of which were contact injuries. We calculated the contact load (collision count and collision load) in addition to distance and high-speed running during the study period. We demonstrated that the collision count and collision load evaluated using EWMA-ACWR showed a positive association with the risk of injury in elite rugby union players. To date, the International Olympic Committee recommends using ACWR to monitor injury risk in many sports (6). If the acute load exceeds the chronic load (i.e., if the acute load increases rapidly and fatigue occurs, or if training in the previous four weeks has been insufficient to improve fitness), it has been reported to increase the risk of injury in various sports (6–10). In these reports, non-contact variables, such as sRPE, overall distance measured by GPS, and distance during high-speed running (27–30), were used as training loads. The sports in these studies were soccer, field hockey, and Gaelic football. Generally, the proportion of contact injuries in sports (31–34) is less than 40%, which is lower than that of rugby union, in which the proportion of contact injuries is more than 75% (20). In rugby union, most injuries were reported to occur during contact play, including tackling (23.0%), being tackled (22.8%), and collision (14.2%) (20), and 70.7% of injuries in the present study were also classified as contact injuries. Therefore, monitoring the injury risk in rugby union should also be considered along with contact load.

This study used EWMA-ACWR to investigate the association between contact load and injury incidence. Although the conventional calculation method of RA-ACWR is easier to calculate, as it equally weighs all load data included in the calculation (35, 36), it has been reported that RA-ACWR is inherently less predictive and introduces statistical artifacts (12, 13). On the other hand, EWMA proposed by Williams et al. (14) is calculated by weighing more recent loads and considering the influence of more recent loads on the occurrence of injury. Several studies have investigated the relationship between match and training load (not contact load, but sRPE, distance, and high-speed running) and injury incidence using RA-ACWR and EWMA-ACWR and showed that EWMA-ACWR was more associated with injury than RA-ACWR (15–18). Therefore, monitoring the EWMA-ACWR of collision load and/or collision count in training and matches might be an effective method to prevent the risk of injury.

This study indicates that the EWMA-ACWR of the contact load measured by GPS devices is associated with the risk of injury in an elite rugby union team. However, this study had some limitations. First, collision metrics were detected by changes in the axis orientation and by an impact force >8 g using a GPS device (STATSports Sonra; STATSports Group Limited, Northern Ireland) and calculated by a weighted algorithm combining the maximum velocity into the collision, peak impact force, and collision duration. However, the details of this algorithm are not available. In addition, this study used position, season, and age as confounders; other risk factors, such as internal load (6), also need to be considered. Furthermore, this study only considered loads that could be measured using GPS devices. Therefore, indoor training, such as gym training, was not included as a training load. Next, because this study evaluated the acute load for 7 days and the chronic load for 28 days, the first 27 measuring days were excluded from the ACWR calculation period. Therefore, it was impossible to assess the first month after the start of training. In addition, the relationship between ACWR and injury may fluctuate when different calculation periods are used for acute and chronic loads (18). Moreover, there is increasing evidence of the limitations of RA-ACWR (12, 13). Similarly, although the EWMA method is commonly used, it is not without its limitations (37, 38). The EWMA-ACWR measurement method is also not standardized; elements such as calculation methods, time-window settings, and analysis methods have not been established. Furthermore, although ACWR has been published in consensus statements by the International Olympic Committee (6) and is widely used worldwide, the consensus statement may be updated in the future. Therefore, future analysis using quantification methods other than EWMA-ACWR (e.g., absolute contact load values and cumulative rolling sums) might be necessary to clarify the relationship between contact load and time-loss injury. Next, this study analyzed the association between ACWR and all injuries during only one season. Future studies need to clarify the relationship between contact load and injury, separated by the nature of the injury (contact/non-contact), severity, and situation (training/match) based on several seasons. Lastly, in the future, conducting intervention studies to determine whether adjustment of contact load reduces the incidence of injury will likely show that contact load is an important factor in injury prevention in rugby union.

In conclusion, the contact load calculated using the EWMA-ACWR was associated with time-loss injury in elite rugby union players. Thus, this study showed as rugby union is a full-contact sport and frequent contact injuries occur, preventing injury in rugby union players also requires monitoring and management of the contact load. Based on this study, coaches and strength and conditioning coaches could possibly make strides in player safety and performance in practice.

## Data Availability

The original contributions presented in the study are included in the article/Supplementary Material, further inquiries can be directed to the corresponding author.
